# Neurocognitive and psychosocial outcomes in survivors of childhood leukemia with Down syndrome

**DOI:** 10.1002/cam4.6842

**Published:** 2024-01-19

**Authors:** Kellen Gandy, Lacey Hall, Kevin R. Krull, Anna J. Esbensen, Jeffrey Rubnitz, Lisa M. Jacola

**Affiliations:** ^1^ Department of Psychology and Biobehavioral Sciences St. Jude Children's Research Hospital Memphis Tennessee USA; ^2^ Department of Social Sciences University of Houston Downtown Houston Texas USA; ^3^ Division of Developmental and Behavioral Pediatrics Cincinnati Children's Hospital Medical Center & University of Cincinnati College of Medicine Cincinnati Ohio USA; ^4^ Department of Oncology St. Jude Children's Research Hospital Memphis Tennessee USA

**Keywords:** childhood ALL, neurocognitive late effects, quality of life, survivorship

## Abstract

**Objective:**

The primary aim of this study was to assess the feasibility of a developmentally tailored neurocognitive assessment in survivors of childhood acute leukemia with Down syndrome (DS‐leukemia). A secondary aim was to compare outcomes in the DS‐leukemia group to a historical comparison group of individuals with DS and no history of childhood cancer.

**Methods:**

Survivors of DS‐leukemia (*n* = 43; 56% male, mean [SD] age at diagnosis = 4.3 [4.5] years; age at evaluation = 15 [7.9] years) completed a neurocognitive assessment battery that included direct measures of attention, executive function, and processing speed, and proxy ratings of attention problems and executive dysfunction. Direct assessment outcomes were compared to a historical comparison cohort of individuals with DS and no history of childhood cancer (DS‐control; *n* = 117; 56% male, mean [SD] age at evaluation = 12.7 [3.4] years).

**Results:**

Rates of valid task completion ranged from 54% to 95%, suggesting feasibility for most direct assessment measures. Compared to the DS‐control group, the DS‐leukemia group had significantly lower completion rates on measures of executive function (*p* = 0.008) and processing speed (*p* = 0.018) compared to the DS‐control group. There were no other significant group differences in completion rates. Compared to the DS‐control group, the DS‐leukemia group had significantly more accurate performance on two measures of executive function (*p* = 0.032; *p* = 0.005). Compared to the DS‐control group, the DS‐leukemia group had significantly more problems with executive function as identified on proxy ratings (6.5% vs. 32.6%, *p* = <0.001).

**Conclusion:**

Children with Down syndrome (DS) are at increased risk for developing acute leukemia compared to the general population but are systematically excluded from neurocognitive outcome studies among leukemia survivors. This study demonstrated the feasibility of evaluating neurocognitive late effects in leukemia survivors with DS using novel measures appropriate for populations with intellectual developmental disorder.

## 1 INTRODUCTION

Acute leukemia is the most common malignancy of childhood, accounting for approximately 25% of all childhood cancers.^1^ Survivors of childhood acute leukemia are at risk for neurocognitive late effects that are associated with reduced functional and quality of life outcomes throughout survivorship.^2^, ^3^, ^4^, ^5^, ^6^ Determinants of neurocognitive problems include younger age at diagnosis, increased treatment intensity, and treatment toxicities, as well as individual and environmental factors.^3^ Given survival rates have increased over time, particularly for childhood acute lymphoblastic leukemia (ALL), and elevated risk for clinically significant neurocognitive impairment, serial neurocognitive monitoring beginning during therapy and continuing into survivorship is a recommended standard of care.^1^, ^7^, ^8^

Down syndrome (DS), trisomy 21, is the most common genetic cause of intellectual disability.^9^ Individuals with DS have a distinct phenotype characterized by atypical neurodevelopment, cognitive impairment, and a heightened susceptibility to medical conditions,^10^ which are important sources of within‐syndrome variability in neurocognitive and functional outcomes.^11^, ^12^ Children with DS and acute myeloid leukemia (AML) constitute 10% of all cases of acute myeloid leukemia and have a 10‐ to 20‐fold excess risk of ALL compared to the general population.^13^, ^14^, ^15^ Compared to children diagnosed with AML without DS, children with DS‐AML have higher survival rates.^16^ Survival prognosis in DS‐ALL is similar or slightly worse when compared to the broader population of children with ALL.^17^ Compared to non‐DS ALL, individuals with DS‐ALL are significantly more likely to experience acute therapy‐related toxicities.^17^, ^18^, ^19^, ^20^ Adult survivors of DS‐leukemia are at significantly greater risk for severe chronic health conditions compared to adult childhood cancer survivors without DS^21^ and experience greater morbidity and mortality than adults with DS and no cancer history.^22^

Neurocognitive outcomes in DS‐leukemia have been excluded from decades of neurocognitive outcome research in survivors of childhood leukemia (e.g., 5, 23–42).^2^, ^5^, ^23^, ^24^, ^25^, ^26^, ^27^, ^28^, ^29^, ^30^, ^31^, ^32^, ^33^, ^34^, ^35^, ^36^, ^37^, ^38^, ^39^, ^40^, ^41^ For example, to date, only one study has reported on neurocognitive outcomes in 43 survivors of DS‐leukemia, including 14 survivors of DS‐ALL.^42^ Compared to an age matched group of 21 children with DS and no cancer history, the DS‐ALL group performed significantly lower on measures of verbal intelligence, academics, vocabulary, and visual‐motor skills, and had significantly poorer caregiver ratings of overall adaptive function. Although differences between patient groups were not statistically compared, performance in the DS‐AML group exceeded the DS‐ALL group in several domains, suggesting that treatment differences have distinct and quantifiable cognitive morbidity, and that ALL therapy is associated with greater impairment.

In a population with DS, a preexisting neurodevelopmental vulnerability, additional skill loss conferred by leukemia and its treatment may critically affect global cognitive and adaptive outcomes.^43^ Reduced opportunities for early intervention, rehabilitative services, and special education services during therapy may further increase neurocognitive risk.^44^, ^45^ Characterization of neurocognitive outcomes in survivors of DS‐leukemia are critical to inform guidelines that are tailored to address the specific profile of neurocognitive late effects in the DS‐ALL population, provide evidence‐based information to families about what to expect following treatment, and identify targets for intervention to improve outcomes.

Traditional neurocognitive measures are not sensitive to variability in performance in lower functioning populations. Developmentally tailored assessment tools are needed to quantify change over time and in response to treatment. The present study examined the feasibility of a novel approach to neurocognitive assessment in survivors of DS‐leukemia. Measures were focused on the cognitive domains of attention, executive function, and processing speed, areas of vulnerability for survivors of childhood leukemia in the general population^3^ and for individuals with DS.^46^ We hypothesized that survivors of DS‐leukemia would have lower completion rates than a historical comparison group of individuals with DS and no history of childhood cancer (DS‐control). A second aim was to characterize neurocognitive outcomes among DS‐leukemia survivors, including comparing outcomes to the DS‐control group. As an exploratory aim, we compared neurocognitive outcomes between DS‐leukemia groups based on diagnosis (DS‐ALL vs. DS‐AML).

## 2 METHODS

### 2.1 Study population

Participants were recruited from the population of survivors of DS‐ALL or DS‐AML that were treated at St. Jude Children's Research Hospital. Survivors were eligible for the study if they were more than 6 months off therapy and English speaking. Survivors were excluded from the study if they had secondary CNS injury or disease (e.g., seizures and stroke) or history of substance abuse. This study was approved by the Institutional Review Board and written informed consent was obtained by the participants and/or their legal guardians.

### 2.2 Neurocognitive assessment

Neurocognitive assessment was completed at one time point. Tests were administered by clinical research associates under the supervision of a board‐certified clinical neuropsychologist (LMJ). Assessments were completed in a single 2.5‐h session. Participants > = 5 years old at study participation completed direct neurocognitive assessment. Caregivers of all participants, regardless of age, completed standardized proxy ratings of attention and executive function (Child Behavior Checklist, CBCL; Behavior Rating Inventory of Executive Function, BRIEF) and adaptive skills (Scales of Independent Behavior Revised, SIB‐R).^47^, ^48^, ^49^

Direct assessment. See Table S1 for details, including specific domains assessed by each measure. The current study reports on the following: measures of attention and executive function (NIH‐Toolbox Flanker Inhibitory Control and Attention and Dimensional Change Card Sort^50^, ^51^; Verbal Fluency,^52^, ^53^, ^54^, ^55^, ^56^ Modified Self Ordered Pointing,^54^, ^55^, ^56^, ^57^, ^58^ Cat‐Dog Stroop,^52^, ^58^ Spatial Reversal,^52^, ^54^, ^59^, ^60^ and Rule Shift Card Task^54^), processing speed (NIH‐Toolbox Pattern Comparison Processing Speed, Cat‐Dog Stroop Naming and Rule Shift Naming), and receptive language (NIH‐Toolbox Picture Vocabulary). Estimated IQ was obtained using a standardized measure to characterize the group on global functioning (KBIT‐II Abbreviated IQ^61^).

### 2.3 Demographic and clinical variables

Demographic variables were obtained using a study questionnaire. Clinical and treatment variables were abstracted from medical records (survivorship care plans). See Table S2 for details of treatment by participant.

### 2.4 Historical comparison

De‐identified data for the DS‐control group (*N* = 117) were obtained from prospective research studies of neurocognitive functioning in individuals with Down syndrome between 2011 and 2018. Participants were recruited through community support groups, research participant registries, and hospital registries. Data were available for direct assessment measures, including Verbal Fluency, Modified Self‐Ordered Pointing, Cat‐Dog Stroop, Rule Shift, and Spatial Reversal, and proxy ratings (BRIEF; CBCL).

### 2.5 Statistical analysis

Descriptive statistics were conducted to characterize groups on demographic, individual, and clinical variables. To examine feasibility (primary aim), task completion rates (% attempted/completed) were calculated for all direct assessment tasks for the DS‐leukemia group and compared between the DS‐leukemia and DS‐control groups using chi‐square.

To characterize neurocognitive outcomes for the DS‐leukemia group (secondary aim), descriptive statistics were calculated for direct assessment and proxy ratings, and outcomes were compared between the DS‐leukemia and DS‐control groups using one‐way ANOVA and chi‐square. To explore differences in neurocognitive outcomes by diagnosis (exploratory aim), univariate comparisons were conducted between the DS‐ALL and the DS‐AML groups, with age at diagnosis and time from treatment included as covariates for the direct assessment measures. Chi‐square was used to examine proxy rating outcomes by diagnosis. All statistical tests were two‐sided with a significance threshold of *p* < 0.05.

Analyses were completed with IBM SPSS Version 28.^62^

## 3 RESULTS

**FIGURE 1:**
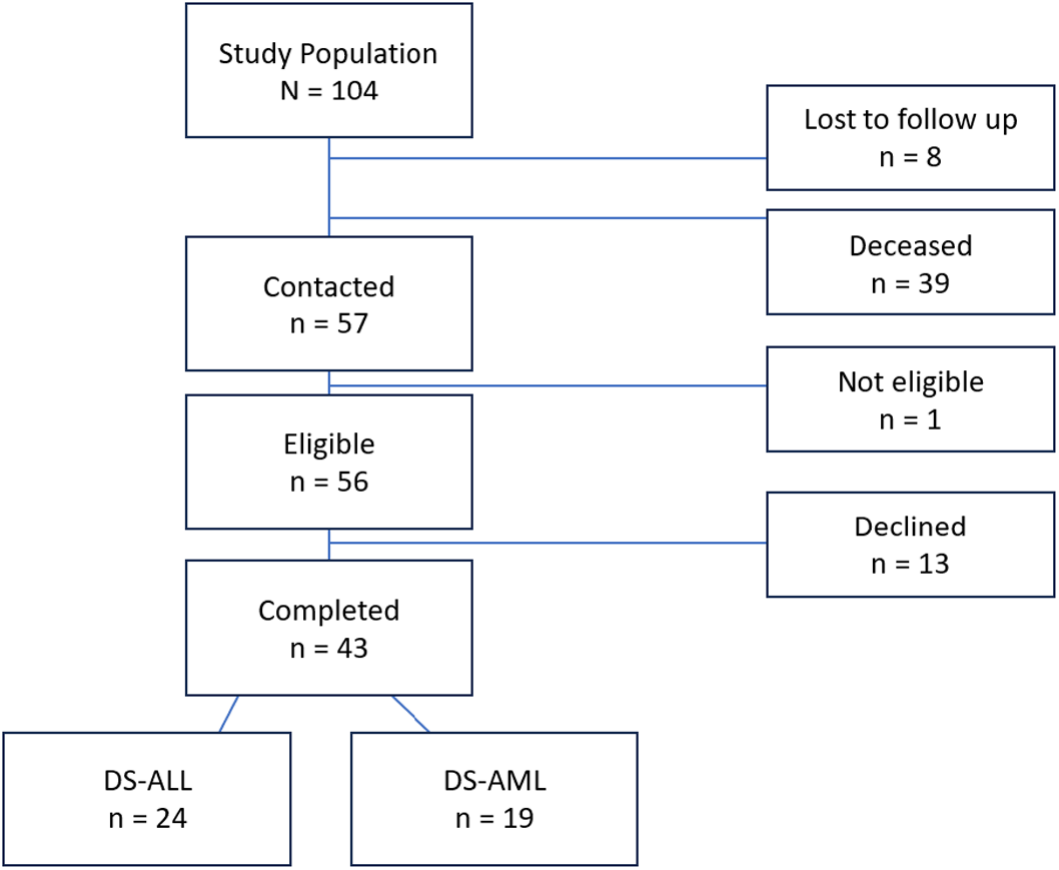
Study flow chart. Study population is survivors of DS‐leukemia treated at St. Jude Children’s Research Hospital between 1980 and 2015.

See Figure 1. A total of 104 individuals with DS diagnosed with ALL or AML (DS‐leukemia) were treated at St. Jude Children's Research Hospital between 1980 and 2015. Of the eligible DS‐leukemia survivors (*n* = 56), 43 (77%) agreed to participate and 13 (23%) declined. Reasons included passive refusal (*n* = 7), unwillingness to travel due to patient's health (*n* = 3), and unwillingness to participate in a neurocognitive study (*n* = 3). Demographic and clinical characteristics are presented in Table 1.

**TABLE 1:** Demographic and clinical characteristics for the DS‐leukemia and DS‐control groups.

		DS‐leukemia						DS‐control	
		Overall group		DS‐ALL		DS‐AML			
		(*n* = 43)		(*n* = 24)		(*n* = 19)		(*n* = 117)	
		*n*	*%*	*n*	*%*	*n*	*%*	*n*	*%*
Sex	Male	24	55.8	13	54.2	11	57.9	65	55.6
	Female	19	44.2	11	45.8	8	42.1	52	44.4
Race/Ethnicity	White	35	81.4	19	79.2	16	84.2	82	70.1
	Black	5	11.6	3	12.5	2	10.5	7	6.0
	Hispanic	2	4.7	2	8.3	0	0.0	1	0.9
	Other	1	2.3	–	–	1	5.3	–	–
	Not reported	–	–	–	–	–	–	27	23.1
Caregiver education	10th–11th grade	–	–	–	–	–	–	4	3.4
	High school graduate	12	27.9	10	41.7	2	10.5	13	11.1
	Some college	13	30.2	5	20.8	8	42.1	29	24.8
	College graduate	12	27.9	5	20.8	7	36.8	31	26.5
	Graduate degree	5	11.6	3	12.5	2	10.5	13	11.1
	Not reported	1	2.3	1	4.2	–	–	27	23.1
Household Income (USD)	< $50,000	12	27.9	7	29.2	5	26.2	14	12.0
	≥ $50,000	25	58.1	13	54.1	12	63.2	17	14.6
	Not reported	6	13.9	4	16.7	2	10.6	86	73.5
		Mean	SD	Mean	SD	Mean	SD	Mean	SD
Age at diagnosis		4.31	4.52	6.72	4.83	1.26	0.50	–	–
Age at evaluation		15.03	7.98	17.52	7.50	11.88	7.60	12.74	3.38
Estimated IQ	KBIT‐II ABIQ (SS)	44.14	7.16	42.21	4.46	48.00	9.85	43.34	5.06
Adaptive skills	SIB‐R global adaptive (SS)	44.24	23.15	38.53	19.84	51.47	25.64	45.84	21.96

*Note*: Standard Scores are age‐standardized with a normative mean of 100 and a standard deviation of 15. Higher scores = better performance or higher ratings of independence.

Abbreviations: ALL, acute lymphoblastic leukemia; AML, acute myeloid leukemia; DS, Down syndrome; KBIT‐2, Kaufman Brief Intelligence Test, Second Edition; SD, standard deviation; SIB‐R, Scales of Independent Behavior, Revised; SS, standard score; USD, United States dollar.

### 3.1 Feasibility of direct assessment measures

See Table 2. Of the 43 total participants, 39 met age‐based criteria for direct neurocognitive assessment. Completion rates for the DS‐leukemia group ranged from 53.8% (Rule Shift 1‐back) to 94.9% (Modified Self Ordered Pointing) and were over 70% for all but two measures (Rule Shift and Cat Dog – Inhibit condition). Compared to the DS‐control group, the DS‐leukemia group had significantly lower completion rates on measures of executive function (Cat Dog ‐ Inhibition; 80.3% vs. 59.0%, *p* = 0.008) and processing speed (Rule Shift ‐ Naming; 86.4% vs. 69.2%, *p* = 0.018) compared to the DS‐control group. There were no other significant group differences in completion rates of direct assessment measures.

**TABLE 2:** Task completion rates for the DS‐leukemia and DS‐control groups.

	DS‐leukemia			DS‐control			*p* (two‐sided)
	Attempted	Completed	Completion rate	Attempted	Completed	Completion rate	
	*n*	*n*	*%*	*n*	*n*	*%*	
Semantic Fluency	32	39	82.1	84	103	81.6	0.945
Phonemic Fluency	29	39	74.4	81	103	78.6	0.586
Modified Self‐Ordered Pointing	37	39	94.9	97	103	94.2	0.872
Cat Dog ‐ Naming	30	39	76.9	99	117	84.6	0.271
Cat Dog ‐ Inhibit	23	39	59.0	94	117	80.3	**0.008**
Rule Shift ‐ Naming	27	39	69.2	89	103	86.4	**0.018**
Rule Shift ‐ 1‐back	21	39	53.8	59	103	57.3	0.713
Spatial Reversal	35	39	89.7	50	52	96.2	0.223
NIH‐TB Flanker Attention	30	39	76.9	–		–	–
NIH‐TB Pattern Comparison	32	39	82.1	–		–	–
NIH‐TB Dimensional Change	30	39	76.9	–		–	–
NIH‐TB Picture Vocabulary	32	39	82.1	–		–	–

*Note*: *p*‐value is from chi‐square comparison of completion rates between the DS‐leukemia and DS‐control groups. Bold font = significant at less *p* < 0.05.

Abbreviations: DS, Down syndrome; NIH‐TB, National Institutes of Health Toolbox.

### 3.2 Neurocognitive outcomes in the DS‐leukemia group

See Table 3. Compared to the DS‐control group, the DS‐leukemia group had significantly more accurate performance on two measures of executive function (Cat Dog Inhibit – Accuracy, 0.72 [0.29], 0.86 [0.20], *p* = 0.032; Rule Shift – Accuracy, 67.15 [18.47], 86.24 [26.72], *p* = 0.005).

**TABLE 3:** Direct assessment scores for the DS‐leukemia and DS‐control groups.

		DS‐leukemia			DS‐control			Mean difference	*p* (2‐sided)
		*n*	Mean	SD	*n*	Mean	SD		
Semantic Fluency	Total correct	33	14.58	10.63	117	16.35	9.33	−1.77	0.200
Phonemic Fluency	Total correct	31	6.74	5.45	81	7.41	4.79	−0.67	0.528
Modified Self‐Ordered Pointing	Total correct	38	21.79	6.12	95	23.07	7.68	−1.28	0.362
Cat Dog ‐ Naming	Accuracy	30	0.98	0.04	99	0.96	0.11	0.02	0.258
	Time (seconds)		22.77	14.73	99	18.09	11.65	4.68	0.073
Cat Dog ‐ Inhibit	Accuracy	22	0.86	0.20	92	0.72	0.29	0.14	**0.032**
	Time (seconds)		34.46	20.74		28.53	12.52	5.93	**0.046**
Spatial Reversal	Accuracy	35	0.68	0.12	50	0.64	0.12	0.04	0.125
Rule Shift ‐ Naming	Accuracy	27	0.96	0.11	90	0.95	0.14	0.01	0.681
	Time (seconds)		48.59	22.84	41	40.37	25.49	8.22	0.180
Rule Shift ‐ 1‐back	Accuracy	21	0.82	0.15	59	0.77	0.17	0.05	0.226
	Time (seconds)		86.24	26.72	27	67.15	18.47	19.09	**0.005**

*Note*: Two‐sided *p*‐value from one‐way ANOVA between the DS‐leukemia and DS‐control groups. Bold font = *p* < 0.05. Higher scores = better performance and slower time.

Abbreviation: SD, standard deviation.

**FIGURE 2:**
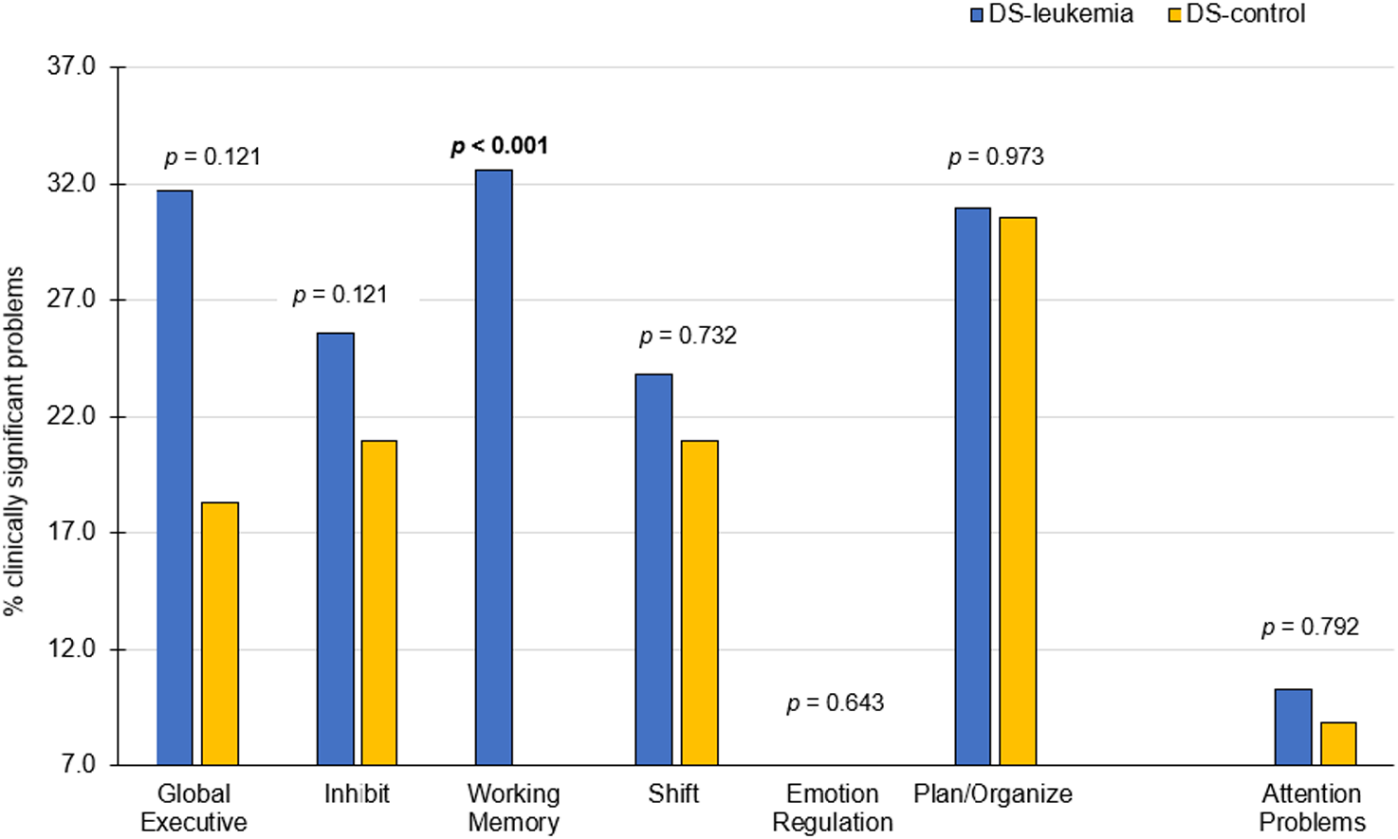
Frequency of caregiver ratings of attention problems and executive dysfunction for the DS‐leukemia and DS‐control groups. BRIEF Scales: Global Executive, Inhibit, Working Memory, Shift, Emotion Regulation, Plan/Organize. CBCL Scale: Attention Problems. Scores are considered clinically significant if they are ≥2 standard deviations above the normative mean, with higher scores indicating more problems. The expected frequency of clinically significant elevations in the normative population is 7%. Two‐sided *p*‐values are from chi‐square frequency comparisons between the DS‐leukemia and DS‐control groups.

Figure 2 depicts the rates of clinically significant elevations of attention problems and executive dysfunction for the DS‐leukemia and DS‐control groups. Compared to normative expectations, the DS‐leukemia group had a significantly greater rate of clinically significant problems with executive function (BRIEF Global Executive Composite, 31.7%, *p* = <0.00001; Inhibit, 25.6%, *p* = 0.0003; Working Memory, 32.6% *p* < 0.0001; Shift, 23.8%, *p* = 0.009; Plan/Organize, 31.0%, *p* < 0.0001). Compared to the DS‐control group, the DS‐leukemia group had significantly more problems with executive function (Working Memory, 6.5% vs. 32.6%, *p* = <0.001).

### 3.3 Exploratory analysis of neurocognitive outcomes by leukemia diagnosis

**FIGURE 3:**
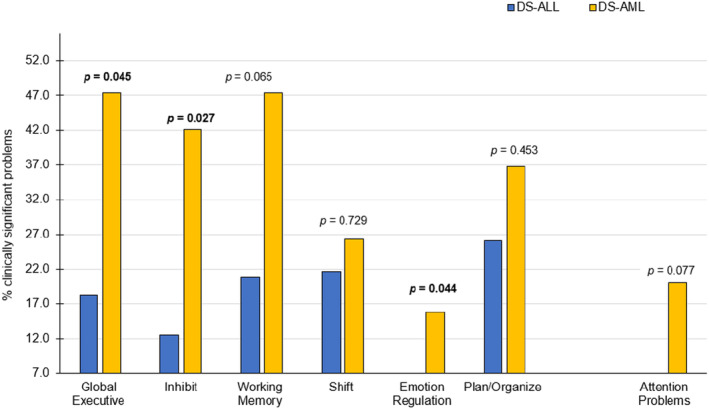
Frequency of caregiver ratings of attention problems and executive dysfunction for the DS‐ALL and DS‐AML groups. BRIEF Scales: Global Executive, Inhibit, Working Memory, Shift, Emotion Regulation, Plan/Organize. CBCL Scale: Attention Problems. Scores are considered clinically significant if they are ≥2 standard deviations above the normative mean, with higher scores indicating more problems. The expected frequency of clinically significant elevations in the normative population is 7%. Two‐sided *p*‐values are from chi‐square frequency comparisons between the DS‐leukemia and DS‐control groups.

See Table S3 and Figure 3. Compared to the DS‐ALL group, the DS‐AML group had a significantly higher frequency of caregiver ratings of executive dysfunction (BRIEF‐Global Executive, 18.2 vs. 47.4%, *p* = 0.045; Inhibit, 12.5 vs. 42.1%, *p* = 0.027; Emotion Regulation, 0.0 vs. 15.8%, *p* = 0.044).

Results from univariate linear regression on performance outcomes (covarying for time from treatment and age at diagnosis) showed significant differences in direct assessment scores on measures of processing speed and executive function. Compared to the DS‐AML group, the DS‐ALL group performed worse on measures of verbal fluency (Semantic Fluency, Estimated Mean [SE], 18.64 [3.08], 12.54 [2.05], *p* = 0.005; Phonemic Fluency, 8.89 [1.65], 5.56 [1.15], *p* = 0.015) and processing speed (NIH‐TB Pattern Comparison, 51.15 [5.51], 35.53 [3.29], *p* = 0.046). Compared to the DS‐ALL group, the DS‐AML group performed worse on a measure of working memory (Self‐Ordered Pointing, 22.77 [1.26], 20.12 [1.75], *p* = 0.041) For all other direct assessment outcomes, performance did not significantly differ between the DS‐ALL and DS‐AML groups.

## 4 DISCUSSION

This study is the first to report on neurocognitive outcomes in survivors of DS‐leukemia using a developmentally tailored assessment. The battery emphasizes measures of attention and executive function, domains that are considered “at‐risk” in childhood leukemia survivors and are disproportionately impaired in individuals with DS. Developmental considerations were emphasized in measurement selection and test administration. For example, Verbal Fluency cues include phonemes that emerge early in speech development in children with DS. Unlike traditional Stroop tasks, the Cat‐Dog Stroop does not require reading. In consideration of the DS phenotype, we selected tests that do not require lengthy verbal responses, and many tests do not require verbal responding. Prior to beginning test administration, each participant is screened for basic abilities necessary to complete certain tests (e.g., the name specific colors for the Rule Shift task).

Our primary aim was to investigate the feasibility of direct assessment in this population. Results provide evidence for feasibility of the direct neurocognitive assessment, with nearly all measures validly completed by >70% of participants and minimal differences in completion rates between the DS‐leukemia and DS‐control groups. These results provide direction for larger, prospective studies of neurocognitive outcomes in survivors of DS‐leukemia, particularly as studies have documented the clinical utility (i.e., developmental sensitivity and predictive validity) of some measures used in the current study in the broader IDD population.^51^, ^63^, ^64^ The feasibility and clinical utility of computerized assessment measures is notable when considering the need for scalability of approach in large, multisite clinical trials.

The secondary aim was to characterize neurocognitive outcomes in survivors of DS‐leukemia. Comparison to the DS‐control group revealed minimal differences in performance outcomes on direct assessment. Specifically, the DS‐leukemia group was slower and more accurate on a measure of inhibition and a measure of working memory. However, it is important to note that these two tasks had the lowest valid completion rates and thus performance is likely not representative of the overall group.

In contrast, results from proxy ratings of participant function, which have documented clinical utility in the DS population,^65^, ^66^, ^67^, ^68^, ^69^ provide evidence for executive dysfunction in the DS‐leukemia group, with a significantly greater number of elevations in working memory problems seen in the DS‐leukemia group compared to the DS‐control group. Although the differences were not statistically significant, the difference in magnitude of clinical elevations of symptoms of global executive function provide further evidence for specific vulnerability in the DS‐leukemia group. Consistent with the recommendations from the outcome measures for clinical trials in Down syndrome working group,^70^ our results suggest that future studies include proxy ratings to best understand the impact of problems in daily life.

Results from the final, exploratory aim showed minimal differences in neurocognitive outcomes by leukemia diagnosis, with significantly worse verbal fluency in the DS‐ALL group compared to the DS‐AML group, and significantly better working memory in the DS‐ALL group compared to the DS‐AML group. Executive functions and processing speed are specific areas of vulnerability in survivors of childhood ALL in the general population, owing to treatment associated alterations in brain structure and function.^71^ The interpretation of the findings of this exploratory analysis is limited by sample size but can be used to inform future studies.

This study is the first to use a developmentally tailored approach to neurocognitive assessment in survivors of DS‐leukemia, a population that is systematically excluded from neurocognitive outcome research in childhood ALL. As a result, the impact of preexisting neurodevelopmental vulnerability has not been considered in clinical practice guidelines that aim to remediate deficits and promote quality of life for survivors. For example, the Psychosocial Standards of Care^8^, ^72^ and the Children's Oncology Group Long Term Follow Up Guidelines^73^ do not consider the added risk conferred by a diagnosis of DS in recommendations for neurocognitive and psychosocial screening. In addition, studies of cognitive intervention in childhood cancer survivors routinely exclude patients with intellectual disability.^74^, ^75^, ^76^, ^77^, ^78^ Thus, the primary strength of the present study is that it serves as a first step toward shifting the paradigm from exclusion to inclusion for this vulnerable group of survivors.

Study limitations provide directions for future research. The present study was retrospective, with heterogeneity in diagnosis and treatment. Some leukemia disease features (e.g., white blood cell count, CNS status, and final risk group) were not consistently available for this retrospective study. Prospective studies of cohorts treated on clinical trials are needed to clarify outcomes and elucidate clinical and treatment risk factors.

This study showed that neurocognitive assessment is feasible and acceptable in leukemia survivors with Down syndrome, which is a population traditionally excluded from childhood cancer survivorship studies. Findings provide direction for larger, prospective studies of neurocognitive outcomes in survivors of childhood ALL, with the ultimate goals of informing guidelines that are tailored to address the specific profile of neurocognitive late effects in survivors with DS‐leukemia, providing evidence‐based information to families about what to expect following treatment, and identifying targets for intervention to improve outcomes. Results from this study may generalize to the significant proportion of childhood cancer survivors with preexisting neurodevelopmental and genetic conditions who are excluded from neurocognitive and functional outcome studies. For example, a recent study revealed that 23% of children with newly diagnosed ALL screened positive for preexisting neurodevelopmental conditions.^79^

## AUTHOR CONTRIBUTIONS

**Kellen Gandy:** Formal analysis (supporting); writing – original draft (lead); writing – review and editing (equal). **Lacey Hall:** Data curation (equal); project administration (equal); writing – review and editing (supporting). **Kevin Krull:** Conceptualization (supporting); funding acquisition (supporting); writing – review and editing (equal). **Anna J. Esbensen:** Conceptualization (supporting); data curation (supporting); funding acquisition (supporting); methodology (supporting). **Jeffrey Rubnitz:** Conceptualization (supporting); funding acquisition (supporting); writing – review and editing (equal). **Lisa Jacola:** Conceptualization (lead); data curation (lead); formal analysis (equal); funding acquisition (lead); methodology (equal); writing – original draft (supporting); writing – review and editing (equal).

## FUNDING INFORMATION

Research support was provided by the National Cancer Institute (P30 CA21765 and GM92666 to St. Jude Children's Research Hospital), the Alex Lemonade Stand Foundation (LMJ), the National Cancer Institute at the National Institutes of Health T32 Institutional Research Training Grant (T32 CA225590 to KRK), the National Institutes of Health (R21 HD082307; R01 HD093754 to AJE), and by the American Lebanese Syrian Associated Charities (ALSAC). The funding source did not have a role in the study design, the collection, analysis, and interpretation of the data, the preparation and writing of the manuscript, or the decision to submit the manuscript for publication.

## CONFLICT OF INTEREST STATEMENT

The authors report no conflict of interest or disclosures.

## Supporting information


**Table S1.**.
Table S2.
Table S3.

## Data Availability

The data supporting this article will be shared upon reasonable request to the corresponding author.
