# Supplementary material for: Neurocognitive and psychosocial outcomes in survivors of childhood leukemia with Down syndrome
Source: Cancer Med. 2024 Jan 19;13(3):e6842. doi: 10.1002/cam4.6842 (PMC10905531; doi:10.1002/cam4.6842)
Supplement: Supplementary file 1 — Table S1.. Table S2. Table S3. [file CAM4-13-e6842-s001.docx]

*Table S1: Neurocognitive direct assessment.*

| Domain | Performance Tasks | Task Descriptions |
| --- | --- | --- |
| Attention/ Executive Function | Verbal Fluency | generate words in response to cues (animals, food/drink, /t/, and /b/) |
|  | Modified Self-Ordered Pointing | point to different pictures on each page in a series without repeating |
|  | Cat-Dog Stroop | name pictured objects (cat and dog); inhibit a well-learned response (say 'dog' when shown a cat picture) |
|  | Spatial Reversal | learn a location rule in order to find a hidden toy |
|  | Rule Shift Card Task | switch between task rules (color naming and 1-back) |
|  | NIHTB Flanker Inhibition and Attention | respond to visual targets while ignoring distractors |
|  | NIHTB Dimensional Change Card Sort | switch between rules for sorting objects |
| Processing Speed | NIHTB Pattern Comparison Processing Speed | speeded decision-making (are objects the same or different) |
| Receptive Language | NIHTB Picture Vocabulary | select a picture from multiple choices that matches a spoken word |

*Table S2:* *Treatment characteristics of leukemia survivors with Down syndrome.*

| \| **ID** \| **Treatment Protocol** \| **Cyclophosphamide IV (mg/m**^2^**)** \| **Cytarabine IV (mg/m**^2^**)** \| **Cytarabine IT (mg)** \| **Daunorubicin IV (mg/m**^2^**)** \| **Doxorubicin IV (mg/m**^2^**)** \| **Etoposide IV (mg/m**^2^**)** \| \| --- \| --- \| --- \| --- \| --- \| --- \| --- \| --- \| \| 1 \| T16 \| 1000 \| 75 \| 325 \| 25 \| 31 \|  \| \| 2 \| T15 \| 3093 \| 2628 \| 1094 \| 51 \| 158 \|  \| \| 3 \| T15 \| 1045 \| 315 \| 1400 \| 61 \| 119 \|  \| \| 4 \| T15 \| 970 \| 604 \| 978 \| 49 \| 60 \|  \| \| 5 \| T16 \| 2189 \| 2185 \| 1698 \| 49 \| 179 \|  \| \| 6 \| NPTP \| 4094 \|  \|  \|  \|  \|  \| \| 7 \| NPTP as per T16 \| 2973 \| 2571 \| 430 \| 50 \| 148 \|  \| \| 8 \| T16 \| 1000 \| 300 \| 1008 \| 51 \| 29 \|  \| \| 9 \| T15 \| 1000 \| 602 \| 853 \| 49 \| 60 \|  \| \| 10 \| NPTP \| 2314 \| 3136 \| 621 \| 100 \|  \|  \| \| 11 \| T16 \| 2242 \| 1824 \| 873 \| 50 \| 138 \|  \| \| 12 \| T15 \| 1000 \| 595 \| 895 \| 51 \| 62 \|  \| \| 13 \| T16 \| 4087 \| 3635 \| 1800 \| 48 \| 183 \|  \| \| 14 \| NPTP \| 3809 \| 3399 \| 1097 \|  \| 208 \|  \| \| 15 \| T15 \| 4509 \| 4265 \| 1067 \| 49 \| 173 \|  \| \| 16 \| T16 \| 1000 \| 529 \| 789 \| 50 \| 30 \|  \| \| 17 \| T13BL \|  \| 1244 \| 867 \| 100 \|  \| 1200 \| \| 18 \| T15 \| 1000 \| 608 \| 330 \| 25 \| 61 \|  \| \| 19 \| T13H \| 9031 \| 8727 \| 595 \| 98 \|  \| 14284 \| \| 20 \| T16 \| 3366 \| 2743 \| 962 \| 49 \| 114 \|  \| \| 21 \| T13BL \|  \| 1209 \| 900 \| 99 \|  \| 1209 \| \| 22 \| NPTP as per CCG1952 \| 1000 \| 922 \|  \|  \| 152 \|  \| \| 23 \| T16 \|  \|  \|  \|  \|  \|  \| \| 24 \| T16 \| 992 \| 446 \| 380 \| 50 \| 30 \|  \| \| 25 \| NPTP as per AML0431 \|  \| 4750 \| 115 \| 144 \|  \| 560 \| \| 26 \| AML0431 \|  \| 2537 \| 171 \| 154 \|  \| 516 \| \| 27 \| NPTP as per AML0431 \|  \| 2459 \| 111 \| 185 \|  \| 315 \| \| 28 \| NPTP as per AML0431 \|  \|  \|  \|  \|  \|  \| \| 29 \| NPTP \|  \| 700 \| 240 \| 84 \|  \| 926 \| \| 30 \| NPTP \|  \| 700 \| 231 \| 90 \|  \| 900 \| \| 31 \| AML97 \|  \| 3216 \| 216 \| 136 \|  \| 989 \| \| 32 \| AML97 \|  \| 700 \| 148 \| 134 \|  \| 1018 \| \| 33 \| NPTP as per POG9241 \|  \| 477 \| 82 \| 93 \|  \| 1023 \| \| 34 \| NPTP \|  \| 2829 \| 44 \| 176 \|  \| 561 \| \| 35 \| NPTP \|  \| 700 \| 268 \| 88 \|  \| 920 \| \| 36 \| NPTP \|  \| 700 \| 231 \| 90 \|  \| 728 \| \| 37 \| AML0431 \|  \| 2847 \| 161 \| 127 \| 56 \| 535 \| \| 38 \| NPTP \|  \| 700 \| 293 \| 83 \|  \| 900 \| \| 39 \| AML0431 \|  \| 2609 \| 130 \| 162 \|  \| 517 \| \| 40 \| AML0431 \|  \| 2866 \| 120 \| 180 \|  \| 555 \| \| 41 \| NPTP as per AML0451 \|  \|  \|  \|  \|  \|  \| \| 42 \| NPTP \|  \| 18430 \| 117 \| 136 \|  \| 811 \| \| 43 \| AML87 \|  \|  \| 261 \| 213 \|  \| 10074 \| |
| --- | --- | --- | --- | --- | --- | --- | --- | --- | --- | --- | --- | --- | --- | --- | --- | --- | --- | --- | --- | --- | --- | --- | --- | --- | --- | --- | --- | --- | --- | --- | --- | --- | --- | --- | --- | --- | --- | --- | --- | --- | --- | --- | --- | --- | --- | --- | --- | --- | --- | --- | --- | --- | --- | --- | --- | --- | --- | --- | --- | --- | --- | --- | --- | --- | --- | --- | --- | --- | --- | --- | --- | --- | --- | --- | --- | --- | --- | --- | --- | --- | --- | --- | --- | --- | --- | --- | --- | --- | --- | --- | --- | --- | --- | --- | --- | --- | --- | --- | --- | --- | --- | --- | --- | --- | --- | --- | --- | --- | --- | --- | --- | --- | --- | --- | --- | --- | --- | --- | --- | --- | --- | --- | --- | --- | --- | --- | --- | --- | --- | --- | --- | --- | --- | --- | --- | --- | --- | --- | --- | --- | --- | --- | --- | --- | --- | --- | --- | --- | --- | --- | --- | --- | --- | --- | --- | --- | --- | --- | --- | --- | --- | --- | --- | --- | --- | --- | --- | --- | --- | --- | --- | --- | --- | --- | --- | --- | --- | --- | --- | --- | --- | --- | --- | --- | --- | --- | --- | --- | --- | --- | --- | --- | --- | --- | --- | --- | --- | --- | --- | --- | --- | --- | --- | --- | --- | --- | --- | --- | --- | --- | --- | --- | --- | --- | --- | --- | --- | --- | --- | --- | --- | --- | --- | --- | --- | --- | --- | --- | --- | --- | --- | --- | --- | --- | --- | --- | --- | --- | --- | --- | --- | --- | --- | --- | --- | --- | --- | --- | --- | --- | --- | --- | --- | --- | --- | --- | --- | --- | --- | --- | --- | --- | --- | --- | --- | --- | --- | --- | --- | --- | --- | --- | --- | --- | --- | --- | --- | --- | --- | --- | --- | --- | --- | --- | --- | --- | --- | --- | --- | --- | --- | --- | --- | --- | --- | --- | --- | --- | --- | --- | --- | --- | --- | --- | --- | --- | --- | --- | --- | --- | --- | --- | --- | --- | --- | --- | --- | --- | --- | --- | --- | --- | --- | --- | --- | --- | --- | --- | --- | --- | --- | --- | --- | --- | --- | --- | --- | --- | --- | --- | --- | --- | --- | --- | --- | --- | --- | --- | --- | --- | --- | --- |

*Table S2 (Continued): Treatment characteristics of leukemia survivors with Down syndrome.*

| **ID** | **High Dose Cytarabine IV (mg/m**^2^**)** | **High Dose**  **Methotrexate**  **IV (mg/m**^2^**)** | **Hydrocortisone**  **IT (mg)** | **L-asparaginase IM (U/m**^2^**)** | **Methotrexate IT (mg)** | **Methotrexate**  **IV (mg/m**^2^**)** | **Thioguanine PO (mg/mg**^2^**)** | **Vincristine IV (mg/m**^2^**)** |
| --- | --- | --- | --- | --- | --- | --- | --- | --- |
| 1 |  | 1475 | 217 |  | 108 | 3263 |  | 42 |
| 2 | 8000 | 1750 | 687 | 543830 | 343 | 1148 |  | 44 |
| 3 |  | 2038 | 619 | 536288 | 310 | 1515 |  | 73 |
| 4 |  | 1964 | 600 | 243846 | 300 | 1649 |  | 66 |
| 5 | 8000 | 2010 | 1132 |  | 566 | 1546 | 59 | 66 |
| 6 |  |  |  |  |  |  |  |  |
| 7 | 3972 |  | 287 | 193975 | 143 | 3681 |  | 49 |
| 8 |  | 2032 | 672 |  | 336 | 3081 | 278 | 68 |
| 9 |  | 2045 | 515 | 253962 | 258 | 3179 |  | 58 |
| 10 |  |  | 414 | 116032 | 207 |  |  | 43 |
| 11 | 8182 | 2004 | 582 |  | 291 | 793 |  | 65 |
| 12 |  | 1982 | 533 | 191091 | 267 | 2576 |  | 58 |
| 13 | 8000 | 2090 | 1200 |  | 600 | 2224 | 656 | 59 |
| 14 | 7907 | 1866 | 731 |  | 366 | 1463 |  | 2511 |
| 15 | 8000 | 2129 | 648 | 530221 | 324 |  |  | 62 |
| 16 |  | 2058 | 526 |  | 263 | 3791 | 647 | 67 |
| 17 |  | 5173 | 578 | 111296 | 289 | 1504 |  | 57 |
| 18 |  | 2020 | 199 | 200470 | 99 | 4070 |  | 40 |
| 19 |  | 4771 | 397 | 119624 | 198 | 1252 |  | 51 |
| 20 | 8046 | 2028 | 642 |  | 321 | 1083 | 506 | 40 |
| 21 |  | 5076 | 600 | 119324 | 300 | 2794 |  | 30 |
| 22 |  |  |  | 68485 | 200 |  | 1623 | 42 |
| 23 |  |  |  |  |  |  |  |  |
| 24 |  | 1759 | 254 |  | 127 | 2739 | 694 | 34 |
| 25 | 18154 |  |  |  |  |  | 769 |  |
| 26 | 15512 |  |  | 7756 |  |  | 707 |  |
| 27 | 18214 |  |  |  |  |  | 889 |  |
| 28 |  |  |  |  |  |  |  |  |
| 29 | 26383 |  |  |  |  |  | 700 |  |
| 30 | 25610 |  |  |  |  |  | 700 |  |
| 31 | 20000 |  |  |  |  |  | 636 |  |
| 32 | 20357 |  |  |  |  |  | 724 |  |
| 33 | 22500 |  |  |  |  |  |  |  |
| 34 | 12767 |  | 29 |  | 15 |  | 995 |  |
| 35 | 26950 |  |  |  |  |  | 700 |  |
| 36 | 26524 |  |  |  |  |  | 571 |  |
| 37 | 16898 |  | 41 | 8571 | 20 |  | . |  |
| 38 | 26000 |  |  |  |  |  | 700 |  |
| 39 | 16089 |  |  | 8044 |  |  | 978 |  |
| 40 | 17490 |  |  | 8760 |  |  | 960 |  |
| 41 |  |  |  |  |  |  |  |  |
| 42 |  |  |  |  |  |  | 672 |  |
| 43 |  |  | 174 |  | 87 |  |  |  |

Abbreviations: IV = intravenous; IT = intrathecal; IM = intramuscular; PO = oral administration. NTPT = non-protocol treatment plan; AML0431 = Children’s Oncology Group AAML0431 clinical trial; AAML0451 = Children’s Oncology Group Clinical Trial - AAML0451; CCG1952 Children’s Oncology Group CCG-1952 clinical trial; POG9241 = Children’s Oncology Group POG9241 clinical trial; T13 = St. Jude Total Therapy Study 13; T15 = St. Jude Total Therapy Study 15; T16 = St. Jude Total Therapy Study 16. AML87 = Leukemia Study Group AML87 clinical trial; AML97 = Leukemia Study Group AML 97 clinical trial.

Notes: Two patients with ALL received cranial radiation therapy and one of these two patients received stem cell transplant.

*Table S3: Completion rates by diagnosis for each of the performance based neurocognitive tasks.*

|  |  |  |  |  |  |  |
| --- | --- | --- | --- | --- | --- | --- |
|  | DS-ALL | | | DS-AML | | |
|  | Attempted | Completed | Completion Rate | Attempted | Completed | Completion Rate |
|  | *n* | *n* | *%* | *n* | *n* | *%* |
| Semantic Fluency | 21 | 24 | 87.5 | 11 | 15 | 73.3 |
| Phonemic Fluency | 18 | 24 | 75.0 | 11 | 15 | 73.3 |
| Modified Self-Ordered Pointing | 23 | 24 | 95.8 | 14 | 15 | 93.3 |
| Cat Dog - Naming | 22 | 24 | 91.7 | 8 | 15 | 53.3 |
| Cat Dog - Inhibit | 16 | 24 | 66.7 | 7 | 15 | 46.7 |
| Rule Shift - Naming | 18 | 24 | 75.0 | 9 | 15 | 60.0 |
| Rule Shift - 1-back | 14 | 24 | 58.3 | 7 | 15 | 46.7 |
| Spatial Reversal | 23 | 24 | 95.8 | 12 | 15 | 80.0 |
| NIH-TB Flanker Attention | 21 | 24 | 87.5 | 9 | 15 | 60.0 |
| NIH-TB Pattern Comparison | 23 | 24 | 95.8 | 9 | 15 | 60.0 |
| NIH-TB Dimensional Change | 18 | 24 | 75.0 | 12 | 15 | 80.0 |
| NIH-TB Picture Vocabulary | 20 | 24 | 83.3 | 12 | 15 | 80.0 |
|  |  |  |  |  |  |  |
|  |  |  |  |  |  |  |
|  |  |  |  |  |  |  |
